# Supplementary material for: Lessons learned about willingness to adopt various protective measures during the early COVID-19 pandemic in three countries
Source: PLoS One. 2022 Mar 29;17(3):e0265892. doi: 10.1371/journal.pone.0265892 (PMC8963567; doi:10.1371/journal.pone.0265892)
Supplement: S1 File — S1 Table: Descriptive Statistics per country; S2 Table: Sample quotas per country; S3 Table: Models 1–2 replicated with experimental condition; S4 Table: Models 3–4 replicated with experimental condition; S5 Table: Model 1 per Country; S6 Table: Model 2 per Country; S7 Table: Model 3 per Country; S8 Table: Model 4 per Country; S1 Fig: Correlation Matrix; Sensitivity analysis. (DOCX) [file pone.0265892.s001.docx]

Additional File 1 to:

**Lessons learned about the willingness to adopt various protective measures during the early COVID-19 pandemic in three countries**

Ana Paula Santana^1 *^, Lars Korn^2,3^, Cornelia Betsch^2,3^, Robert Böhm^4,1,5^

^1^ Department of Psychology, University of Copenhagen, Denmark

^2^ Media and Communication Science, University of Erfurt, Germany

^3^ Centre for Empirical Research in Economics and Behavioural Sciences (CEREB), University of Erfurt, Germany

^4^ Faculty of Psychology, University of Vienna, Austria

^5^ Copenhagen Centre for Social Data Science (SODAS), University of Copenhagen, Denmark

* Corresponding author: [apss@psy.ku.dk](mailto:apss@psy.ku.dk)

**Contents**

**Tables and Figures**

Table S1. Descriptive Statistics per country Page 3

Table S2. Sample quotas per country Page 4

Table S3. Models 1-2 replicated with experimental condition Page 5

Table S4. Models 3-4 replicated with experimental condition Page 6

Table S5. Model 1 per Country Page 6

Table S6. Model 2 per Country Page 9

Table S7. Model 3 per Country Page 11

Table S8. Model 4 per Country Page 12

Figure S1. Correlation Matrix Page 14

**Sensitivity Analysis** Page 15

**Table S1.** Descriptive Statistics per country

| Variables | Germany (N = 333) | HongKong (N = 367) | USA (N = 495) |
| --- | --- | --- | --- |
| Willingness to adopt the measures |  |  |  |
| mean (sd) | 6.03 ± 0.99 | 5.60 ± 0.92 | 6.10 ± 1.03 |
| Perceived Effectiveness |  |  |  |
| mean (sd) | 5.91 ± 1.00 | 5.57 ± 0.93 | 5.95 ± 1.03 |
| Past Experience with the measures |  |  |  |
| mean (sd) | 4.96 ± 1.60 | 5.07 ± 1.27 | 4.64 ± 1.57 |
| Prosocial Motivation |  |  |  |
| mean (sd) | 6.08 ± 1.09 | 5.70 ± 0.94 | 6.22 ± 1.08 |
| Selfish Motivation |  |  |  |
| mean (sd) | 5.98 ± 1.03 | 5.63 ± 0.96 | 6.08 ± 1.14 |
| Collectivism |  |  |  |
| mean (sd) | 5.25 ± 0.85 | 5.10 ± 0.81 | 5.52 ± 0.90 |
| Individualism |  |  |  |
| mean (sd) | 4.71 ± 0.80 | 4.81 ± 0.78 | 4.69 ± 0.82 |

*Note.* Regarding levels of collectivism and individualism across countries, an exploratory one-way ANOVA showed that the countries differed in levels of collectivism, *F*(2, 1192) = 21.46, *p* < .001, but not in levels of individualism, *F*(2, 1192) = 2.061, *p* < .128.

**Table S2.** Sample quotas per country

| Country | Gender | Quota ID | Age | Target | Count |
| --- | --- | --- | --- | --- | --- |
| US | M | 1 | 18-24 | 21 | 32 |
|  |  | 2 | 25-34 | 28 | 28 |
|  |  | 3 | 35-44 | 27 | 27 |
|  |  | 4 | 45-54 | 31 | 31 |
|  |  | 5 | 55-64 | 25 | 73 |
|  |  | 6 | 65+ | 27 | 111 |
|  | F | 7 | 18-24 | 22 | 46 |
|  |  | 8 | 25-34 | 30 | 30 |
|  |  | 9 | 35-44 | 30 | 30 |
|  |  | 10 | 45-54 | 32 | 32 |
|  |  | 11 | 55-64 | 26 | 26 |
|  |  | 12 | 65+ | 29 | 29 |
| HK | M | 1 | 18-24 | 18 | 30 |
|  |  | 2 | 25-34 | 29 | 29 |
|  |  | 3 | 35-44 | 34 | 35 |
|  |  | 4 | 45-54 | 32 | 34 |
|  |  | 5 | 55-64 | 18 | 24 |
|  |  | 6 | 65+ | 23 | 25 |
|  | F | 7 | 18-24 | 20 | 26 |
|  |  | 8 | 25-34 | 32 | 34 |
|  |  | 9 | 35-44 | 38 | 39 |
|  |  | 10 | 45-54 | 37 | 44 |
|  |  | 11 | 55-64 | 21 | 21 |
|  |  | 12 | 65+ | 26 | 26 |
| GER | M | 1 | 18-24 | 15 | 16 |
|  |  | 2 | 25-34 | 23 | 23 |
|  |  | 3 | 35-44 | 27 | 28 |
|  |  | 4 | 45-54 | 28 | 28 |
|  |  | 5 | 55-64 | 26 | 26 |
|  |  | 6 | 65+ | 39 | 39 |
|  | F | 7 | 18-24 | 16 | 16 |
|  |  | 8 | 25-34 | 24 | 24 |
|  |  | 9 | 35-44 | 29 | 29 |
|  |  | 10 | 45-54 | 31 | 31 |
|  |  | 11 | 55-64 | 28 | 30 |
|  |  | 12 | 65+ | 42 | 43 |

**Table S3.** Models 1-2 replicated with experimental condition

|  |  | Model S1 |  |  | Model S2 |  |
| --- | --- | --- | --- | --- | --- | --- |
| *Predictors* | *β* | *CI* | *p* | *β* | *CI* | *p* |
| Intercept | 5.99 | 5.93, 6.06 | <.001 | 6.02 | 5.96, 6.09 | <.001 |
| Treatment [75%] | -0.06 | -0.11, 0.00 | 0.061 | -0.05 | -0.11, 0.00 | 0.071 |
| Effectiveness | 0.24 | 0.22, 0.26 | <.001 | 0.23 | 0.21, 0.26 | <.001 |
| Experience | 0.07 | 0.06, 0.08 | <.001 | 0.08 | 0.07, 0.10 | <.001 |
| Intended other-protection | 0.26 | 0.24, 0.28 | <.001 | 0.23 | 0.21, 0.26 | <.001 |
| Intended self-protection | 0.27 | 0.25, 0.29 | <.001 | 0.26 | 0.23, 0.28 | <.001 |
| Collectivism | 0.03 | -0.00, 0.07 | 0.070 | 0.05 | 0.01, 0.08 | .012 |
| Individualism | -0.01 | -0.05, 0.02 | 0.432 | -0.02 | -0.06, 0.02 | 0.286 |
| Country[HongKong] | -0.16 | -0.24, -0.08 | <.001 | -0.18 | -0.26, -0.11 | <.001 |
| Country [USA] | 0.01 | -0.06, 0.08 | 0.810 | 0.02 | -0.05, 0.09 | 0.624 |
| Intended other-protection*Collectivism | 0.02 | 0.00, 0.04 | .016 | 0.04 | 0.02, 0.07 | <.001 |
| Intended other-protection*Individualism | -0.02 | -0.04, -0.00 | .017 | -0.05 | -0.07, -0.03 | <.001 |
| Effectiveness*Experience |  |  |  | -0.01 | -0.02, -0.00 | .007 |
| Effectiveness*Intended other-protection |  |  |  | -0.01 | -0.02, 0.00 | 0.156 |
| Effectiveness*Intended self-protection |  |  |  | 0.03 | 0.01, 0.04 | <.001 |
| Effectiveness*Collectivism |  |  |  | -0.05 | -0.07, -0.03 | <.001 |
| Effectiveness*Individualism |  |  |  | 0.05 | 0.02, 0.07 | <.001 |
| Experience*Intended other-protection |  |  |  | -0.02 | -0.03, -0.01 | .002 |
| Experience*Intended self-protection |  |  |  | -0.03 | -0.03, -0.02 | <.001 |
| Experience*Collectivism |  |  |  | 0.01 | -0.00, 0.02 | 0.222 |
| Experience*Individualism |  |  |  | 0.01 | -0.01, 0.02 | 0.377 |
| Random Effects | | | | | | |
| σ^2^ | 0.68 | | | 0.66 | | |
| τ_00_ | 0.16 _ID_ | | | 0.16 _ID_ | | |
| ICC | 0.19 | | | 0.20 | | |
| N | 1195 _ID_ | | | 1195 _ID_ | | |
| Observations | 8365 | | | 8365 | | |
| Marginal R^2^ / Conditional R^2^ | 0.553 / 0.640 | | | 0.560 / 0.646 | | |

**Table S4.** Models 3-4 replicated with experimental condition

|  | Model S3 | | | Model S4 | | |
| --- | --- | --- | --- | --- | --- | --- |
| *Predictors* | *β* | *CI* | *p* | *β* | *CI* | *p* |
| Intercept | 6.06 | 5.98, 6.13 | **<.001** | 6.13 | 6.05, 6.20 | **<.001** |
| Treatment [75%] | -0.05 | -0.12, 0.02 | 0.189 | -0.04 | -0.11, 0.03 | 0.264 |
| Effectiveness | 0.48 | 0.46, 0.50 | **<.001** | 0.45 | 0.43, 0.47 | **<.001** |
| Experience | 0.12 | 0.10, 0.13 | **<.001** | 0.12 | 0.10, 0.13 | **<.001** |
| PMI [Prosocial] | -0.25 | -0.31, -0.19 | **<.001** | -0.35 | -0.41, -0.29 | **<.001** |
| Collectivism | 0.12 | 0.08, 0.17 | **<.001** | 0.13 | 0.08, 0.17 | **<.001** |
| Individualism | -0.02 | -0.07, 0.02 | 0.331 | -0.02 | -0.07, 0.03 | 0.385 |
| Country:HongKong | -0.26 | -0.35, -0.16 | **<.001** | -0.30 | -0.39, -0.20 | **<.001** |
| Country:USA | 0.04 | -0.05, 0.13 | 0.352 | 0.04 | -0.05, 0.13 | 0.392 |
| Experience:Effectiveness |  |  |  | -0.07 | -0.07, -0.06 | **<.001** |
| PMI [Prosocial]:Effectiveness |  |  |  | -0.16 | -0.20, -0.12 | **<.001** |
| Collectivism:Effectiveness |  |  |  | -0.02 | -0.04, 0.00 | 0.089 |
| Individualism:Effectiveness |  |  |  | 0.02 | -0.00, 0.04 | 0.101 |
| Experience:PMI [Prosocial] |  |  |  | 0.03 | 0.00, 0.06 | **.042** |
| Experience:Collectivism |  |  |  | 0.01 | -0.00, 0.02 | 0.114 |
| Experience:Individualism |  |  |  | 0.00 | -0.01, 0.02 | 0.803 |
| Random Effects | | | | | | |
| σ^2^ | 0.80 | | | 0.76 | | |
| τ_00_ | 0.27 _ID_ | | | 0.27 _ID_ | | |
| ICC | 0.25 | | | 0.26 | | |
| N | 1195 _ID_ | | | 1195 _ID_ | | |
| Observations | 8365 | | | 8365 | | |
| Marginal R^2^ / Conditional R^2^ | 0.405 / 0.556 | | | 0.423 / 0.576 | | |

**Table S5.** Model 1 per Country

|  | Model 1 - Germany | | | Model 1 - Hong Kong | | | Model 1 - United States | | |
| --- | --- | --- | --- | --- | --- | --- | --- | --- | --- |
| *Predictors* | *β* | *CI* | *p* | *β* | *CI* | *p* | *β* | *CI* | *p* |
| Intercept | 0.00 | -0.04, 0.04 | <.001 | -0.00 | -0.04, 0.05 | <.001 | -0.00 | -0.03, 0.03 | <.001 |
| Effectiveness | 0.29 | 0.25, 0.33 | <.001 | 0.23 | 0.19, 0.26 | <.001 | 0.20 | 0.17, 0.23 | <.001 |
| Experience | 0.08 | 0.05, 0.11 | <.001 | 0.12 | 0.08, 0.15 | <.001 | 0.12 | 0.10, 0.14 | <.001 |
| IOP | 0.26 | 0.22, 0.31 | <.001 | 0.18 | 0.15, 0.23 | <.001 | 0.27 | 0.24, 0.31 | <.001 |
| ISP | 0.22 | 0.18, 0.26 | <.001 | 0.21 | 0.17, 0.25 | <.001 | 0.34 | 0.31, 0.38 | <.001 |
| Collectivism | 0.03 | -0.01, 0.07 | 0.176 | 0.16 | 0.11, 0.22 | <.001 | 0.04 | 0.00, 0.07 | 0.059 |
| Individualism | -0.02 | -0.06, 0.02 | 0.376 | -0.05 | -0.10, 0.00 | 0.047 | -0.03 | -0.07, 0.00 | 0.060 |
| IOP x Collectivism | 0.01 | -0.01, 0.04 | 0.360 | 0.00 | -0.05, 0.04 | 0.849 | -0.01 | -0.03, 0.02 | 0.507 |
| IOP x Individualism | -0.04 | -0.07, -0.01 | 0.014 | -0.02 | -0.06, 0.02 | 0.372 | 0.01 | -0.01, 0.04 | 0.294 |
| Random Effects | | | | | | | | | |
| σ^2^ | 0.66 | | | 0.68 | | | 0.67 | | |
| τ_00_ | 0.15 _ID_ | | | 0.14 _ID_ | | | 0.19 _ID_ | | |
| ICC | 0.19 | | | 0.17 | | | 0.22 | | |
| N | 333 _ID_ | | | 367 _ID_ | | | 495 _ID_ | | |
| Observations | 2331 | | | 2569 | | | 3465 | | |
| Marginal R^2^ / Conditional R^2^ | 0.567 / 0.649 | | | 0.438/ 0.535 | | | 0.598 / 0.688 | | |

*Note*. Mixed effects model (prediction of willingness to adopt protective measures [1 - 7]): All predictors were centered at their mean. Standardized coefficients are reported here. IOP = intended other-protection, ISP = intended self-protection.

**Table S6.** Model 2 per Country

|  | Model 2 - Germany | | | Model 2 - Hong Kong | | | Model 2 - United States | | |
| --- | --- | --- | --- | --- | --- | --- | --- | --- | --- |
| *Predictors* | *β* | *CI* | *p* | *β* | *CI* | *p* | *β* | *CI* | *p* |
| Intercept | 0.02 | -0.02, 0.06 | <.001 | -0.01 | -0.06, 0.03 | <.001 | 0.02 | -0.01, 0.06 | <.001 |
| Effectiveness | 0.26 | 0.22, 0.30 | <.001 | 0.24 | 0.20, 0.28 | <.001 | 0.20 | 0.17, 0.23 | <.001 |
| Experience | 0.09 | 0.06, 0.12 | <.001 | 0.12 | 0.08, 0.16 | <.001 | 0.14 | 0.11, 0.16 | <.001 |
| IOP | 0.26 | 0.21, 0.30 | <.001 | 0.16 | 0.12, 0.21 | <.001 | 0.23 | 0.19, 0.27 | <.001 |
| ISP | 0.21 | 0.17, 0.25 | <.001 | 0.21 | 0.17, 0.25 | <.001 | 0.32 | 0.28, 0.36 | <.001 |
| Collectivism | 0.03 | -0.01, 0.08 | 0.135 | 0.17 | 0.12, 0.23 | <.001 | 0.05 | 0.02, 0.09 | 0.006 |
| Individualism | -0.02 | -0.06, 0.02 | 0.281 | -0.06 | -0.11, -0.01 | 0.026 | -0.04 | -0.07, 0.00 | 0.042 |
| Experience x Effectiveness | -0.06 | -0.09, -0.02 | 0.004 | 0.04 | 0.00, 0.07 | 0.027 | -0.05 | -0.08, -0.02 | 0.001 |
| IOP x Effectiveness | -0.04 | -0.07, -0.01 | 0.020 | -0.00 | -0.04, 0.03 | 0.944 | 0.00 | -0.02, 0.03 | 0.904 |
| ISP x Effectiveness | 0.05 | 0.02, 0.08 | <.001 | 0.04 | 0.01, 0.07 | 0.023 | 0.03 | 0.01, 0.06 | 0.009 |
| Collectivism x Effectiveness | -0.07 | -0.11, -0.04 | <.001 | -0.01 | -0.06, 0.03 | 0.517 | -0.03 | -0.07, -0.01 | 0.028 |
| Individualism x Effectiveness | 0.00 | -0.04, 0.04 | 0.938 | 0.00 | -0.04, 0.04 | 0.965 | 0.05 | 0.02, 0.08 | 0.002 |
| IOP x Experience | -0.01 | -0.05, 0.02 | 0.478 | -0.04 | -0.07, 0.00 | 0.049 | -0.04 | -0.07, -0.01 | 0.019 |
| ISP x Experience | -0.05 | -0.08, -0.01 | 0.018 | -0.03 | -0.06, 0.01 | 0.132 | -0.06 | -0.09, -0.03 | <.001 |
| Collectivism x Experience | -0.02 | -0.06, 0.01 | 0.220 | 0.03 | -0.01, 0.06 | 0.194 | 0.03 | 0.00, 0.06 | 0.045 |
| Individualism x Experience | 0.02 | -0.02, 0.06 | 0.305 | -0.01 | -0.05, 0.03 | 0.683 | 0.01 | -0.02, 0.04 | 0.635 |
|  | Model 2 - Germany | | | Model 2 - Hong Kong | | | Model 2 - United States | | |
| *Predictors* | *β* | *CI* | *p* | *β* | *CI* | *p* | *β* | *CI* | *p* |
| IOP x Collectivism | 0.07 | 0.04, 0.11 | <.001 | -0.02 | -0.06, 0.04 | 0.611 | -0.01 | -0.04, 0.03 | 0.751 |
| IOP x Individualism | -0.04 | -0.08, 0.00 | 0.032 | -0.01 | -0.05, 0.04 | 0.739 | -0.02 | -0.05, 0.02 | 0.351 |
| Random Effects | | | | | | | | | |
| σ^2^ | 0.63 | | | 0.68 | | | 0.65 | | |
| τ_00_ | 0.14 _ID_ | | | 0.14 _ID_ | | | 0.20 _ID_ | | |
| ICC | 0.19 | | | 0.17 | | | 0.23 | | |
| N | 333 _ID_ | | | 367 _ID_ | | | 495 _ID_ | | |
| Observations | 2331 | | | 2569 | | | 3465 | | |
| Marginal R^2^ / Conditional R^2^ | 0.584 / 0.661 | | | 0.441 / 0.538 | | | 0.604 / 0.696 | | |

*Note*. Mixed effects model (prediction of willingness to adopt protective measures [1 - 7]): All predictors were centered at their mean. Standardized coefficients are reported here. IOP = intended other-protection, ISP = intended self-protection.

**Table S7.** Model 3 per Country

|  | Model 3 - Germany | | | Model 3 - Hong Kong | | | Model 3 - United States | | |
| --- | --- | --- | --- | --- | --- | --- | --- | --- | --- |
| *Predictors* | *β* | *CI* | *p* | *β* | *CI* | *p* | *β* | *CI* | *p* |
| Intercept | 0.03 | -0.02, 0.07 | <.001 | 0.03 | -0.02, 0.08 | <.001 | 0.04 | 0.00, 0.08 | <.001 |
| Effectiveness | 0.56 | 0.53, 0.60 | <.001 | 0.33 | 0.29, 0.36 | <.001 | 0.48 | 0.45, 0.51 | <.001 |
| Experience | 0.12 | 0.09, 0.16 | <.001 | 0.18 | 0.14, 0.21 | <.001 | 0.19 | 0.16, 0.22 | <.001 |
| PMI[Prosocial] | -0.14 | -0.22, -0.06 | <.001 | -0.13 | -0.20, -0.06 | <.001 | -0.26 | -0.33, -0.20 | <.001 |
| Collectivism | 0.09 | 0.04, 0.14 | <.001 | 0.25 | 0.19, 0.32 | <.001 | 0.13 | 0.08, 0.17 | <.001 |
| Individualism | -0.05 | -0.10, -0.01 | 0.022 | -0.08 | -0.14, -0.02 | 0.007 | -0.04 | -0.08, 0.01 | 0.084 |
| Random Effects | | | | | | | | | |
| σ^2^ | 0.75 | | | 0.71 | | | 0.86 | | |
| τ_00_ | 0.21 _ID_ | | | 0.28 _ID_ | | | 0.30 _ID_ | | |
| ICC | 0.22 | | | 0.28 | | | 0.26 | | |
| N | 333 _ID_ | | | 367 _ID_ | | | 495 _ID_ | | |
| Observations | 2331 | | | 2569 | | | 3465 | | |
| Marginal R^2^ / Conditional R^2^ | 0.468 / 0.583 | | | 0.284 / 0.483 | | | 0.427/ 0.574 | | |

*Note.* PMI (Protection Motivation Index): for each measure, intention to protect oneself was subtracted from the intention to protect others. PMI values greater than zero were classified as prosocial, and pro-self otherwise. Brackets indicate the category of the varible.

**Table S8.** Model 4 per Country

|  | Model 4 - Germany | | | Model 4, Hong Kong | | | Model 4, United States | | |
| --- | --- | --- | --- | --- | --- | --- | --- | --- | --- |
| *Predictors* | *β* | *CI* | *p* | *β* | *CI* | *p* | *β* | *CI* | *p* |
| Intercept | 0.08 | 0.03, 0.13 | <.001 | 0.02 | -0.03, 0.08 | <.001 | 0.10 | 0.06, 0.14 | <.001 |
| Effectiveness | 0.51 | 0.47, 0.55 | <.001 | 0.37 | 0.32, 0.41 | <.001 | 0.42 | 0.39, 0.46 | <.001 |
| Experience | 0.13 | 0.09, 0.16 | <.001 | 0.16 | 0.12, 0.20 | <.001 | 0.19 | 0.16, 0.22 | <.001 |
| PMI[Prosocial] | -0.21 | -0.29, -0.13 | <.001 | -0.16 | -0.23, -0.09 | <.001 | -0.35 | -0.43, -0.28 | <.001 |
| Collectivism | 0.08 | 0.04, 0.13 | 0.001 | 0.25 | 0.19, 0.31 | <.001 | 0.14 | 0.09, 0.18 | <.001 |
| Individualism | -0.06 | -0.10, -0.01 | 0.019 | -0.08 | -0.14, -0.02 | 0.013 | -0.04 | -0.08, 0.01 | 0.104 |
| Experience x Effectiveness | -0.13 | -0.16, -0.10 | <.001 | 0.01 | -0.02, 0.04 | 0.423 | -0.19 | -0.22, -0.16 | <.001 |
| PMI[Prosocial] x Effectiveness | -0.14 | -0.21, -0.07 | <.001 | -0.13 | -0.20, -0.06 | <.001 | -0.17 | -0.23, -0.11 | <.001 |
| Collectivism x Effectiveness | -0.02 | -0.06, 0.01 | 0.131 | 0.00 | -0.05, 0.04 | 0.865 | -0.04 | -0.07, -0.01 | 0.007 |
| Individualism x Effectiveness | -0.05 | -0.08, -0.01 | 0.014 | 0.00 | -0.04, 0.05 | 0.875 | 0.04 | 0.01, 0.07 | 0.004 |
| PMI[Prosocial] x Experience | 0.02 | -0.06, 0.10 | 0.696 | 0.02 | -0.05, 0.10 | 0.502 | 0.03 | -0.03, 0.10 | 0.310 |
| Collectivism x Experience | -0.03 | -0.06, 0.01 | 0.139 | 0.02 | -0.02, 0.06 | 0.254 | 0.03 | 0.00, 0.06 | 0.030 |
| Individualism x Experience | 0.04 | 0.00, 0.08 | 0.031 | -0.03 | -0.07, 0.02 | 0.246 | 0.00 | -0.03, 0.03 | 0.876 |
| Random Effects |  | | |  | | |  | | |
| σ^2^ | 0.71 | | | 0.71 | | | 0.79 | | |
| τ_00_ | 0.21 _ID_ | | | 0.27 _ID_ | | | 0.30 _ID_ | | |
| ICC | 0.23 | | | 0.27 | | | 0.27 | | |
| N | 333 _ID_ | | | 367 _ID_ | | | 495 _ID_ | | |
| Observations | 2331 | | | 2569 | | | 3465 | | |
| Marginal R^2^ / Conditional R^2^ | 0.490 / 0.608 | | | 0.293 / 0.487 | | | 0.462 / 0.608 | | |

*Note.* PMI (Protection Motivation Index): for each measure, intention to protect oneself was subtracted from the intention to protect others. PMI values greater than zero were classified as prosocial, and pro-self otherwise. Brackets indicate the category of the varible.


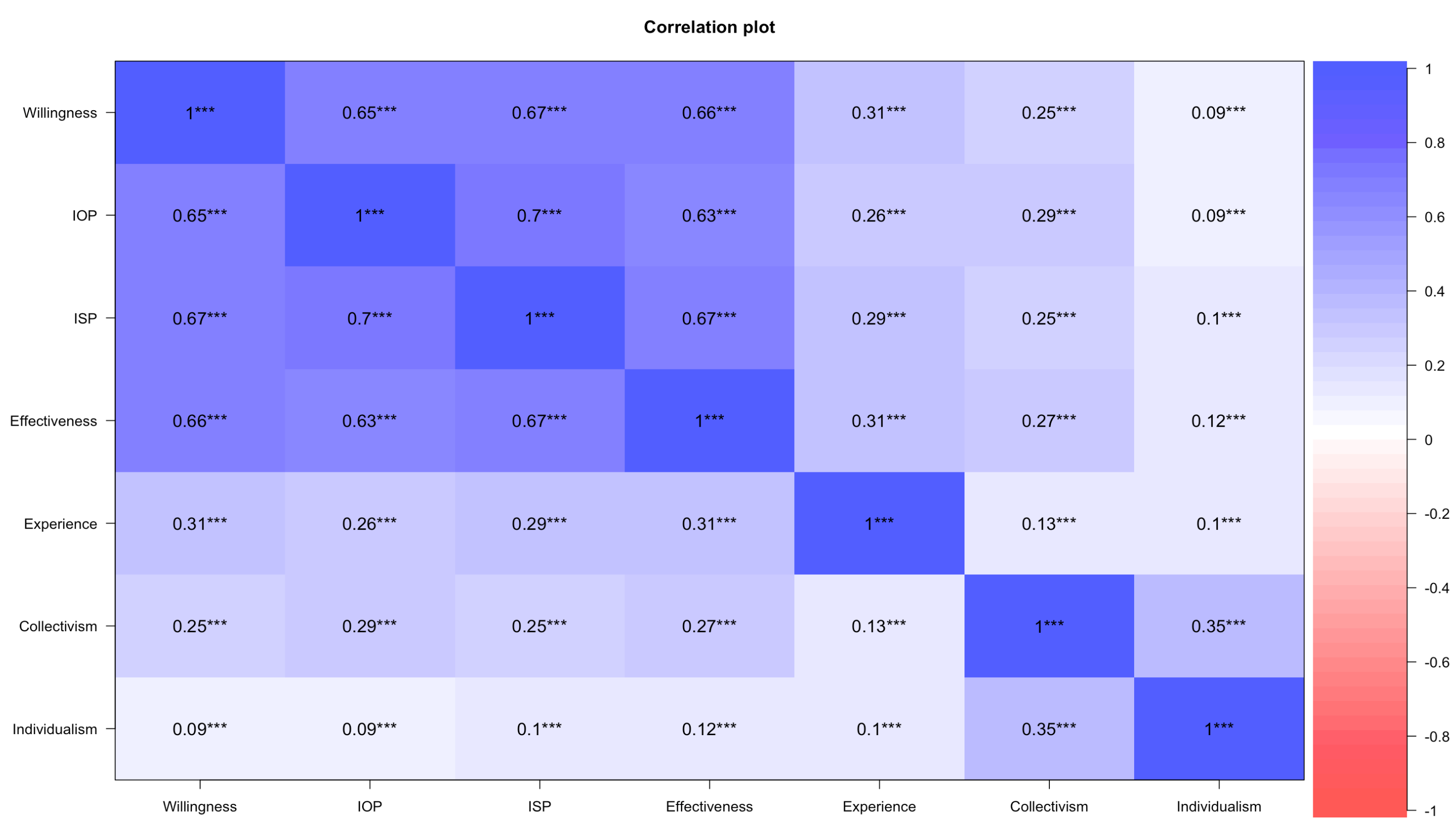


**Figure S1.** Correlation matrix including predictors and outcome (willingness). IOP = intended other-protection, ISP = intended self-protection.

**Sensitivity Analysis**

In order to test achieved power, a sensitivity power analysis was conducted to detect the effect size from the mixed-effects regressions reported in the study. We simulated data using the R package ‘simr’ based on the model testing our main hypotheses (Model 1). Considering the skewness of predictors’ distributions, we assumed beta distributions for all variables. Since collectivism and individualism were scales, decimal numbers were allowed. The other variables were measured separately for each protective measure on a scale from 1 to 7. Therefore, beta distribution was restricted to integers between 1 and 7. Two simulations were performed testing different effect sizes. The power to detect an effect size of 0.02, based on the interaction between intended other-protection and collectivism (hypothesis 3), was .72, 95% CI [.68, .76] (500 simulations). For an effect size of 0.2, based on the main effect of effectiveness (hypothesis 1), the power was 1, CI [99.26, 100.0] (500 simulations). Code for this analysis is available online via the Open Science Framework (OSF) at https://osf.io/6drph/?view_only=eeae5576e5af4e17a6df9acd6ce1d8cd.
